# Epidemiology and genetic variation of acute viral gastroenteritis in children under five years in the Middle East (2020–2025): a systematic review and meta-analysis

**DOI:** 10.1186/s12879-026-13412-5

**Published:** 2026-05-09

**Authors:** Amira M. Zakaria, Samar M. El Shahidy, Atef M. Diab

**Affiliations:** 1 Institute of Biotechnology for Postgraduate Studies and Research, Suez Canal University, Ismailia, Egypt; 2 Faculty of Science, Suez Canal University, Ismailia, Egypt

**Keywords:** Acute gastroenteritis, Rotavirus, Norovirus, Genotype, Middle East, Children under five, Meta-analysis, Molecular epidemiology

## Abstract

**Background:**

Acute viral gastroenteritis remains a major cause of morbidity and hospitalization among children under five years worldwide. In the Middle East, epidemiological and molecular evidence remains fragmented, particularly in the post-COVID-19 period. This systematic review and meta-analysis aimed to synthesize recent data (2020–2025) on the prevalence, genetic diversity, and co-infection patterns of enteric viruses among children aged 0–59 months in the region.

**Methods:**

A comprehensive search of PubMed/MEDLINE, Scopus, Web of Science, ScienceDirect, and the WHO Global Index Medicus identified eligible observational and molecular studies published between 1 January 2020 and 31 May 2025. Study selection, data extraction, and risk-of-bias assessment were independently conducted by two reviewers according to PRISMA 2020 guidelines. The protocol was prospectively registered in PROSPERO (CRD420251064184). Pooled prevalence estimates were calculated using a random-effects model with Freeman–Tukey double arcsine transformation.

**Results:**

Forty-three studies, including 22,021 children tested for acute gastroenteritis (AGE) from nine Middle Eastern countries, met the inclusion criteria. Rotavirus (28 studies) was the most prevalent pathogen, with a pooled prevalence of 30.4% (95% CI: 24.3–35.8; I² = 96%, *p* < 0.001), followed by norovirus (12 studies) at 23.5% (95% CI: 11.4–29), adenovirus (13 studies) at 11.3% (95% CI: 8.6–17.6), and astrovirus (10 studies) at 6.0% (95% CI: 1.3–12.7). Predominant rotavirus genotypes included G1, G2, G3, and G9, commonly combined with P[8], P[4], and P[6], with G3P[8] and G1P[8] as dominant constellations. Norovirus GII.4 and recombinant GII.4[P16] strains were frequently detected. Viral co-infections were also reported, particularly involving rotavirus and other enteric viruses.

**Conclusion:**

Rotavirus and norovirus remain the principal viral causes of pediatric acute gastroenteritis in the Middle East and exhibit substantial genetic diversity with frequent co-infection patterns. However, marked inter-study heterogeneity and uneven geographic representation limit regional generalizability. Strengthened molecular surveillance, standardized diagnostic approaches, and continuous genotype monitoring are essential to optimize prevention strategies and vaccination policies across the region.

**Supplementary Information:**

The online version contains supplementary material available at 10.1186/s12879-026-13412-5.

## Introduction

Acute viral gastroenteritis remains a major cause of illness and hospitalization among children under five worldwide [1]. In the Middle East, the disease burden persists despite advances in sanitation, healthcare access, and the introduction of rotavirus vaccines in several national immunization programs [2]. Countries in the region, including Iran, Egypt, Lebanon, Saudi Arabia, and others, continue to report substantial morbidity, particularly in low- and middle-income settings. Rotavirus, norovirus, adenovirus, astrovirus, and sapovirus are the predominant viral agents implicated in pediatric gastroenteritis [2]. These viruses exhibit marked genetic diversity, affecting transmissibility, pathogenicity, and vaccine performance. Notably, emergent norovirus variants such as GII.4 Sydney and GII.17 Kawasaki have been identified in regional surveillance, including in Iran and Lebanon, underscoring the need for continued molecular monitoring [2, 3].

The COVID-19 pandemic (post-2020) further altered the landscape of viral gastroenteritis through changes in healthcare utilization, hygiene behavior, and viral transmission dynamics [4]. These shifts may have influenced the prevalence and genetic profiles of circulating enteric viruses. Although molecular tools like RT-PCR, genotyping, and next-generation sequencing are increasingly utilized in the region, data remain fragmented and inconsistently reported [5]. To date, no comprehensive review has synthesized post-pandemic evidence on the molecular epidemiology of viral gastroenteritis in children under five across the Middle East.

The primary objective of this systematic review and meta-analysis was to estimate the prevalence and distribution of major viral pathogens causing acute gastroenteritis among children under five years in the Middle East. Secondary objectives included describing circulating viral genotypes and assessing reported patterns of viral co-infection. For this review, the Middle East was defined as comprising 16 countries: Egypt, Iran, Iraq, Israel, Jordan, Kuwait, Bahrain, Lebanon, Oman, Palestine, Qatar, Saudi Arabia, Turkey, Syria, the United Arab Emirates, and Yemen. However, eligible data were available from only nine countries, which should be considered when interpreting regional representativeness. This review advances previous work by focusing on recent evidence published between January 2020 and May 2025, restricting the analysis to children under five years of age, providing a comprehensive synthesis of viral genotypic diversity, and patterns of viral co-infection within the post-COVID-19 epidemiological context.

## Methods

### Study protocol

This systematic review and meta-analysis were conducted following the Preferred Reporting Items for Systematic Reviews and Meta-Analyses (PRISMA) 2020 guidelines [6]. We prospectively registered the protocol in the PROSPERO registry *(CRD420251064184).*

### Search-strategy

A comprehensive literature search was performed across five electronic databases: PubMed/MEDLINE, Scopus, Web of Science, ScienceDirect, and WHO Global Index Medicus, to identify relevant studies published between 1 January 2020 and 31 May 2025. The search targeted original articles reporting on the prevalence and molecular characteristics of viral gastroenteritis pathogens, including rotavirus, norovirus, adenovirus, astrovirus, sapovirus, and bocavirus, among children under five years in Middle Eastern countries. The search strategy combined Medical Subject Headings (MeSH) and free-text terms. Keywords included: (“gastroenteritis” OR “diarrhea” OR “enteric infection”) AND (“virus” OR “viral” OR “rotavirus” OR “norovirus” OR “adenovirus” OR “astrovirus” OR “sapovirus” OR “bocavirus”) AND (“molecular epidemiology” OR “genotyping” OR “phylogenetic” OR “genetic diversity”) AND (“children” OR “pediatric” OR “infants” OR “under five”) AND (“Middle East” OR “Saudi Arabia” OR “Egypt” OR “Iran” OR “Jordan” OR “Lebanon” OR “Qatar” OR “Kuwait” OR “Iraq” OR “Yemen” OR “Syria” OR “Bahrain” OR “Oman” OR “UAE” OR “Palestine” OR “Turkey” OR “Israel”). A detailed search strategy is available in the supplement file.

### Eligibility-criteria

Eligible studies were conducted in 16 Middle Eastern countries: The United Arab Emirates (UAE), Qatar, Kuwait, Bahrain, Oman, Yemen, Iraq, Syria, Lebanon, Jordan, Palestine, Egypt, Iran, Israel, Saudi Arabia, and Turkey, and included children aged 0–59 months. These countries are geographically illustrated in Fig. 3, which presents the regional distribution of the included studies. Studies addressing enteric viral infections (rotavirus, norovirus, adenovirus, astrovirus, or sapovirus) and reporting epidemiological (e.g., prevalence, incidence) and/or molecular data (e.g., genotyping, phylogenetic analysis) were considered. Observational (cross-sectional, cohort, case-control), surveillance, and molecular epidemiological studies published in English or Arabic between 1 January 2020 and 31 May 2025, were eligible. Mixed-age studies were included only when data for the 0–59-month subgroup could be clearly disaggregated; otherwise, they were excluded. Studies were further excluded if they lacked extractable prevalence or molecular data, focused exclusively on bacterial or parasitic pathogens, fell outside the predefined geographic or temporal scope, or had inaccessible full texts. No strict clinical case definition was imposed, and studies reporting any clinical manifestations consistent with acute gastroenteritis were included regardless of hospitalization status or whether infections were community- or hospital-acquired.

### Study selection and data extraction

All retrieved records were organized and deduplicated before screening using Zotero software (Zotero, Roy Rosenzweig Center for History and New Media). Titles and abstracts were screened independently by two reviewers using the Rayyan web application for systematic reviews [7]. Full texts of potentially eligible studies were evaluated against the inclusion criteria. Inconsistencies were addressed by consensus or third-reviewer input. For included studies, data were extracted using a predesigned form that captured: study characteristics (authors, year, country, setting, study design), sample size, study period, diagnostic methods, virus type, genotype distribution, and co-infection rates. This ensured a consistent and comprehensive synthesis of epidemiological and molecular data across studies.

### Risk of bias assessment

The methodological quality of the included studies was evaluated through a risk of bias assessment. Risk of bias was independently assessed by two reviewers using the Joanna Briggs Institute (JBI) Critical Appraisal Checklists to ensure methodological consistency and rigor. Nine methodological domains were evaluated for each study: sample representativeness, adequacy of sample size, clarity of sampling method, validity of measurement instruments, standardization of data collection procedures, control of confounding variables, appropriateness of statistical analysis, adequacy of response rates/missing data management, and clarity of reporting. Detailed assessment criteria for each domain are provided in the Supplementary Material. Each domain was rated as “Yes,” “No,” or “Unclear,” and an overall risk-of-bias classification was assigned based on the proportion of domains rated “Yes”: low risk (≥ 70%), moderate risk (50–69%), and high risk (< 50%). Disagreements between reviewers were resolved through discussion and consensus. Studies classified as high risk were excluded in sensitivity analyses to evaluate the robustness of the pooled estimates.

### Meta-analysis and synthesis of results

Study-specific prevalence estimates were first calculated for each included study, along with their corresponding 95% confidence intervals (95% CI). To stabilize the variance of proportions before pooling, the Freeman–Tukey double arcsine transformation was applied. Pooled prevalence estimates were then computed using a random-effects model, given the anticipated between-study variability related to differences in study populations, geographic settings, and diagnostic methodologies. The between-study variance (τ²) was estimated as part of the random-effects model. Statistical heterogeneity was assessed using Cochran’s Q statistic and quantified using the I² statistic and τ² values. Forest plots were generated to visually present individual study estimates alongside the pooled prevalence estimates and their corresponding 95% CIs. All analyses were conducted using the Data Analysis ToolPak and the Real Statistics Resource Pack add-in implemented in Microsoft Excel (Microsoft Corp., Redmond, WA, USA), following previously validated Excel-based meta-analysis approaches [8]. 

### Terminology standardization

In this review, the term *“Rotavirus”* was used throughout the manuscript to refer to rotavirus infections reported in the included studies. Although most human infections correspond to *Rotavirus A*, several studies reported only rotavirus genotypes (G and P types) without explicitly specifying the viral species. Therefore, the broader term *“Rotavirus”* was adopted to ensure consistency and to encompass all reported genotypes across the included studies.

## Results

### Database search and screening

**Fig. 1:**
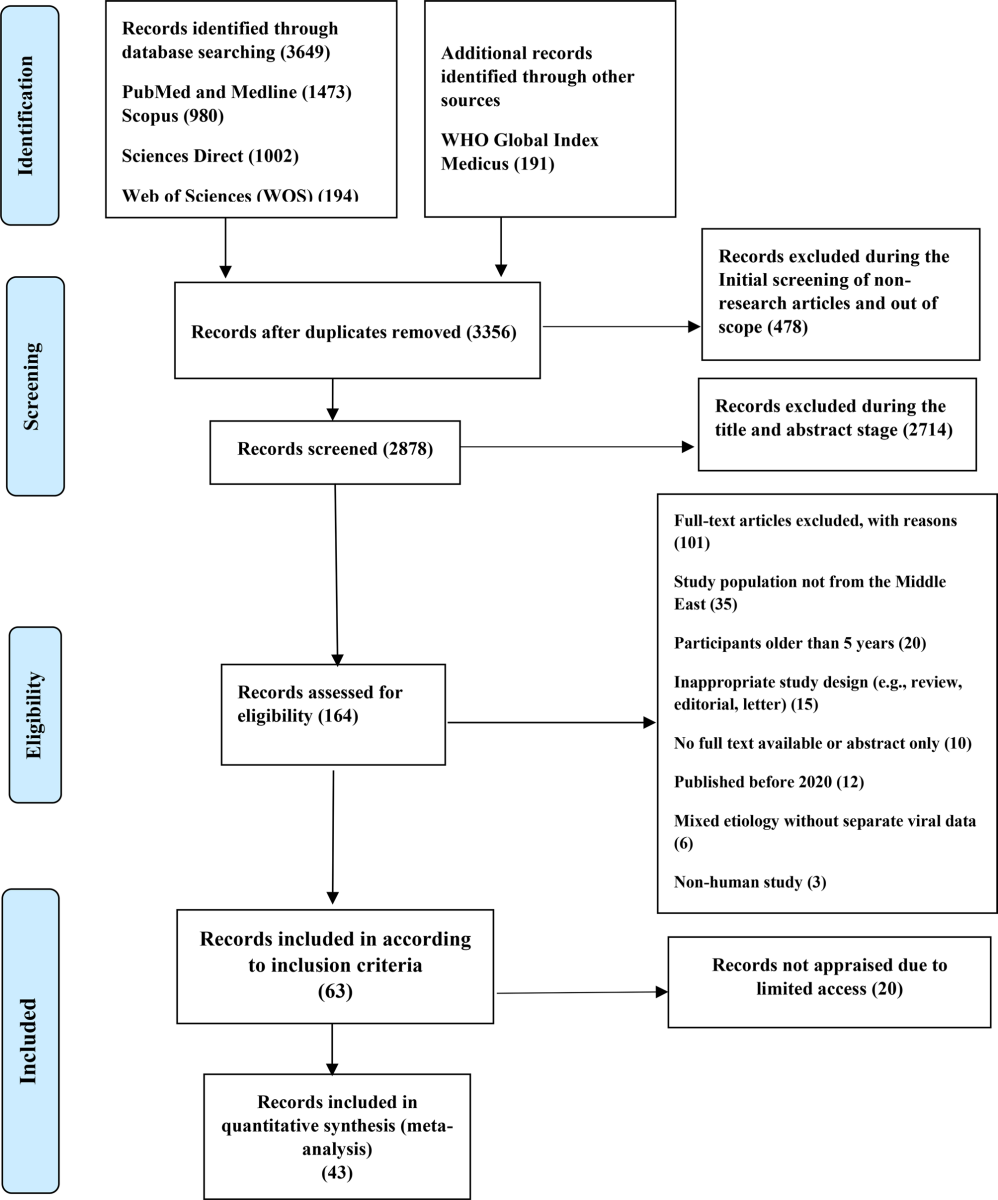
PRISMA flow diagram illustrating the study selection process, including the stages of identification, screening, eligibility, and inclusion. The diagram presents the number of records retrieved from databases, duplicates removed, records screened, full-text articles assessed, exclusions at each stage, and the final number of studies included in the analysis

A total of 3,649 records were identified through electronic database searching, including PubMed/Medline (1,473), Scopus (980), ScienceDirect (1002), and Web of Science (194), with an additional 191 records identified through the WHO Global Index Medicus. After removing duplicates, 3,356 records remained for screening. Following automated filtering, records were reduced from 3356 to 2878 after exclusion of non-research article types (e.g., editorials, letters, and conference abstracts) and studies clearly outside the scope of the review, including those lacking extractable prevalence or molecular data, addressing non-viral pathogens, falling outside the predefined geographic or temporal scope, or with inaccessible full texts. During title and abstract screening, 2714 records were excluded for not meeting the eligibility criteria, primarily because they did not focus on viral gastroenteritis in children under five years of age or involved irrelevant populations. The remaining 164 records underwent full-text assessment, of which 101 studies were further excluded based on predefined criteria, including study population, participant age, study design, publication date, outcome relevance, or unavailability of full text despite attempts to retrieve articles through author contact and institutional repositories (20). Ultimately, 43 studies were included in the final quantitative synthesis and meta-analysis (Fig. 1).

### Characteristics of included studies

**Fig. 2:**
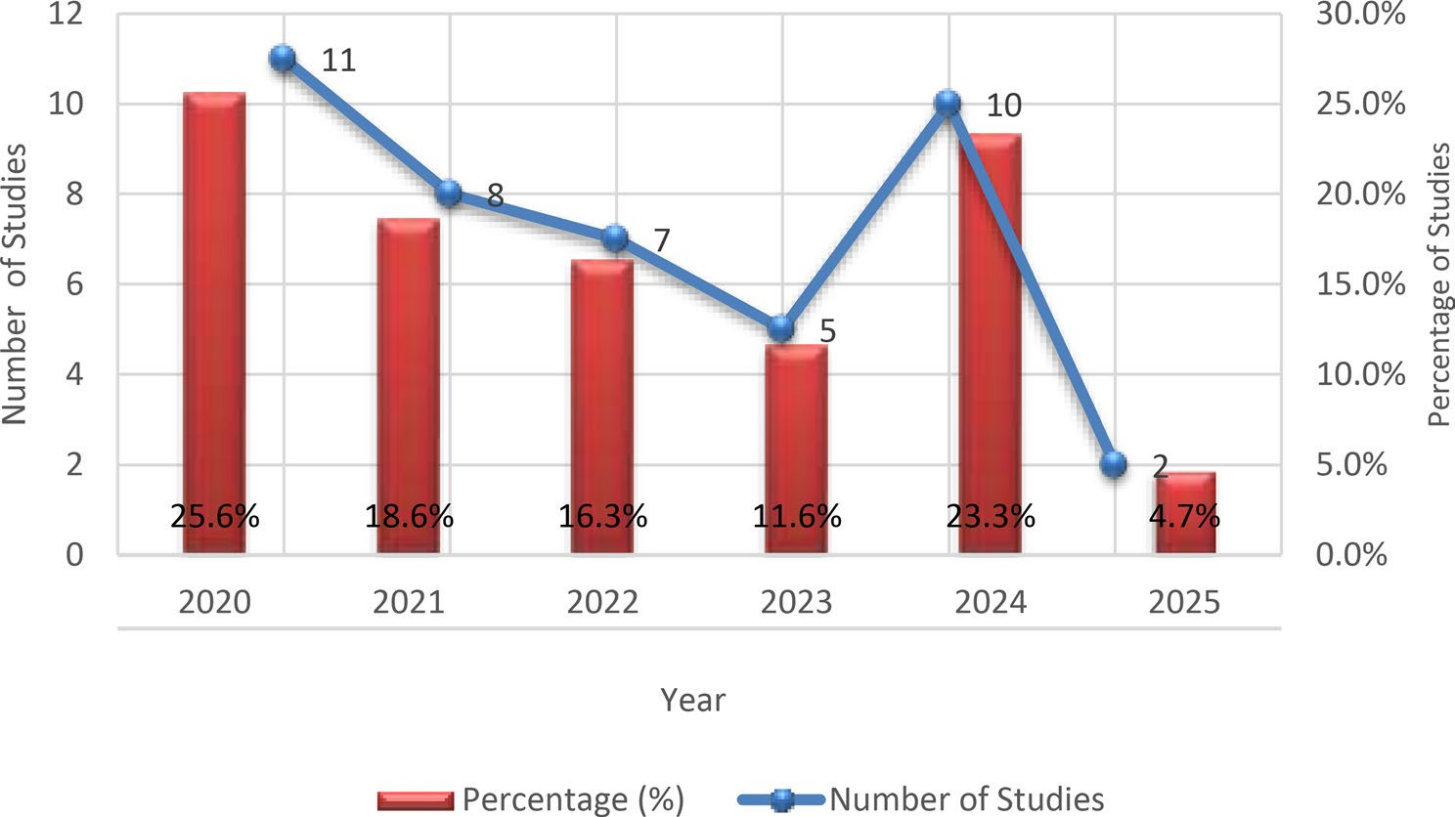
Annual distribution of included studies (2020–2025). Red bars represent the percentage contribution of studies published each year relative to the total included studies (*n* = 43). The blue line indicates the absolute number of studies published per year. The figure demonstrates temporal variation in publication output, with peak contributions observed in 2020 and 2024, followed by a marked decline in 2025

**Fig. 3:**
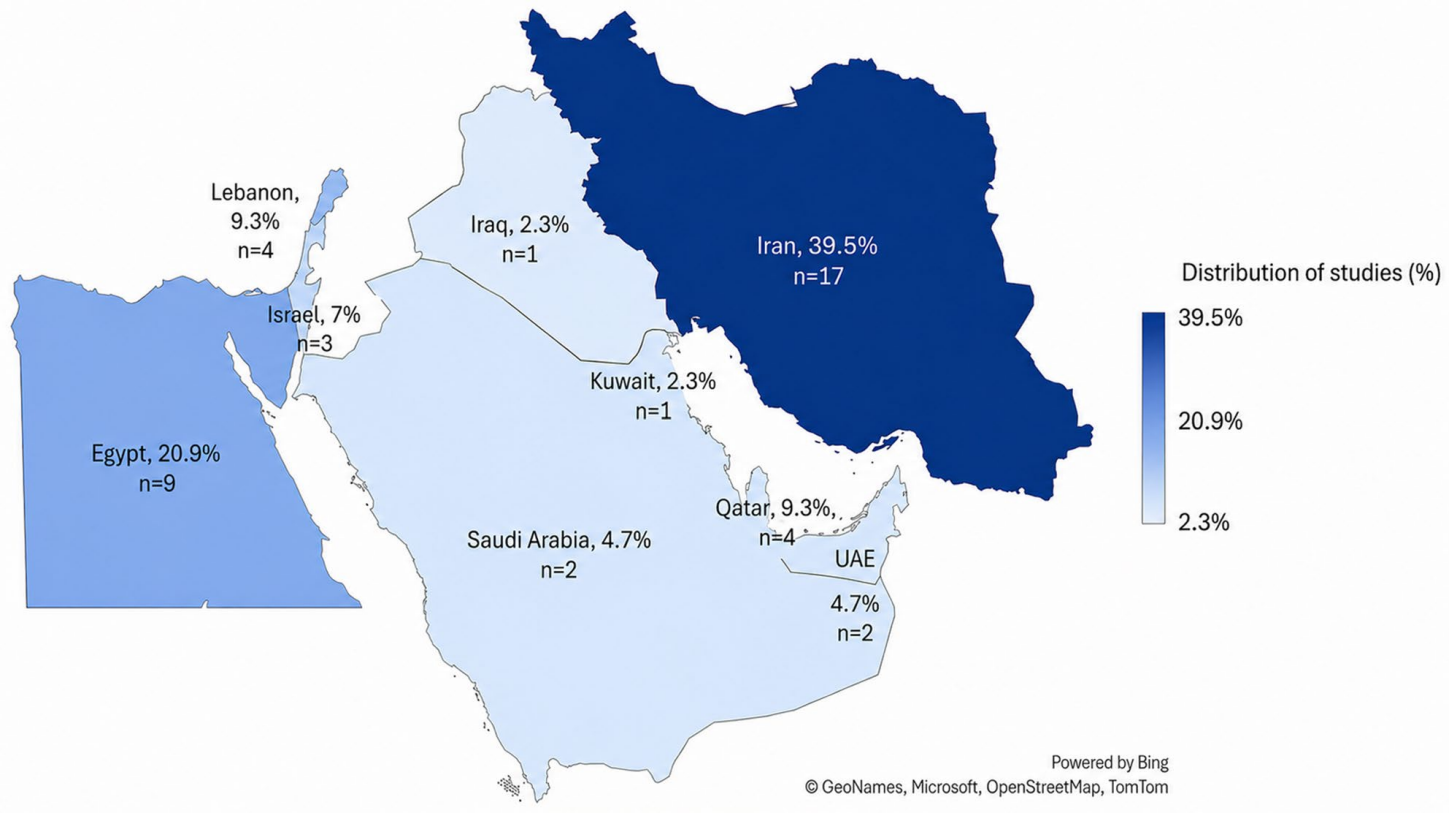
Geographic distribution of included studies in the Middle East (2020–2025). Countries are shaded according to their proportional contribution to the total included studies (*n* = 43), with darker colors indicating higher representation. The value “n” denotes the number of studies per country

Characteristics of included studies investigating viral etiology of acute gastroenteritis in children in the Middle East (2020–2025) are illustrated in Table 1. Detailed characteristics and findings of the included studies are provided in Supplementary Table 2. A total of 43 studies published between 2020 and 2025 were included in the final synthesis. The highest number of publications was recorded in 2020 (11; 25.6%) and 2024 (10; 23.3%), followed by 2021 (8; 18.6%), 2022 (7; 16.3%), 2023 (5; 11.6%), and 2025 (2; 4.7%), Fig. 2. Geographically, although the Middle East comprises 16 countries, only nine were represented in the eligible studies. Iran contributed the largest share (17; 39.5%), followed by Egypt (9; 20.9%). Lebanon and Qatar each contributed four studies (9.3%), while Israel accounted for three (7.0%). The United Arab Emirates and Saudi Arabia were each represented by two studies (4.7%), and Kuwait and Iraq contributed one study each (2.3%). No eligible studies from the remaining seven Middle Eastern countries were identified during the review period. The geographical distribution of the included studies is presented in Fig. 3.

Cross-sectional studies were the most prevalent, accounting for 29 studies (67.4%), and were primarily conducted to estimate the prevalence of viral gastroenteritis among pediatric populations. Retrospective observational studies were the second most common design (four studies), mainly addressing temporal and epidemiological trends. Analytical designs were limited, with only two case–control studies identified. Prospective research was scarce, comprising two prospective studies and one longitudinal observational study. In addition, approximately nine studies focused on molecular or genomic analyses, including viral genotyping and sequencing, whereas only a limited number employed metagenomic sequencing for broader viral detection and characterization. No randomized or interventional studies were identified among the included literature. Fecal specimens were the predominant biological samples collected across studies, serving as the standard medium for viral detection in children with acute gastroenteritis. Overall, the included studies were largely descriptive, with limited representation of higher-level analytical or interventional research designs.

Regarding diagnostic approaches, molecular detection techniques were the most widely used across the included studies. PCR-based methods, including RT-PCR, nested PCR, and multiplex PCR, represented the dominant diagnostic tools for viral detection due to their high sensitivity and specificity. These techniques were particularly prevalent in studies conducted in Iran, Egypt, Israel, and the United Arab Emirates. Advanced diagnostic platforms were also reported in studies from Qatar and Kuwait, where multiplex panels, Film Array systems, and metagenomic sequencing were applied. Sequencing-based methods, including Sanger sequencing and whole genome sequencing, were mainly utilized in genomic surveillance studies, particularly in research conducted in Lebanon and Iran, to characterize viral strains and assess molecular diversity. Immunoassay techniques such as ELISA and rapid antigen detection tests were less frequently used and were more common in earlier studies, mainly in reports from Egypt, Saudi Arabia, and Iraq.

### Study-specific prevalence and genotypic diversity

**Fig. 4:**
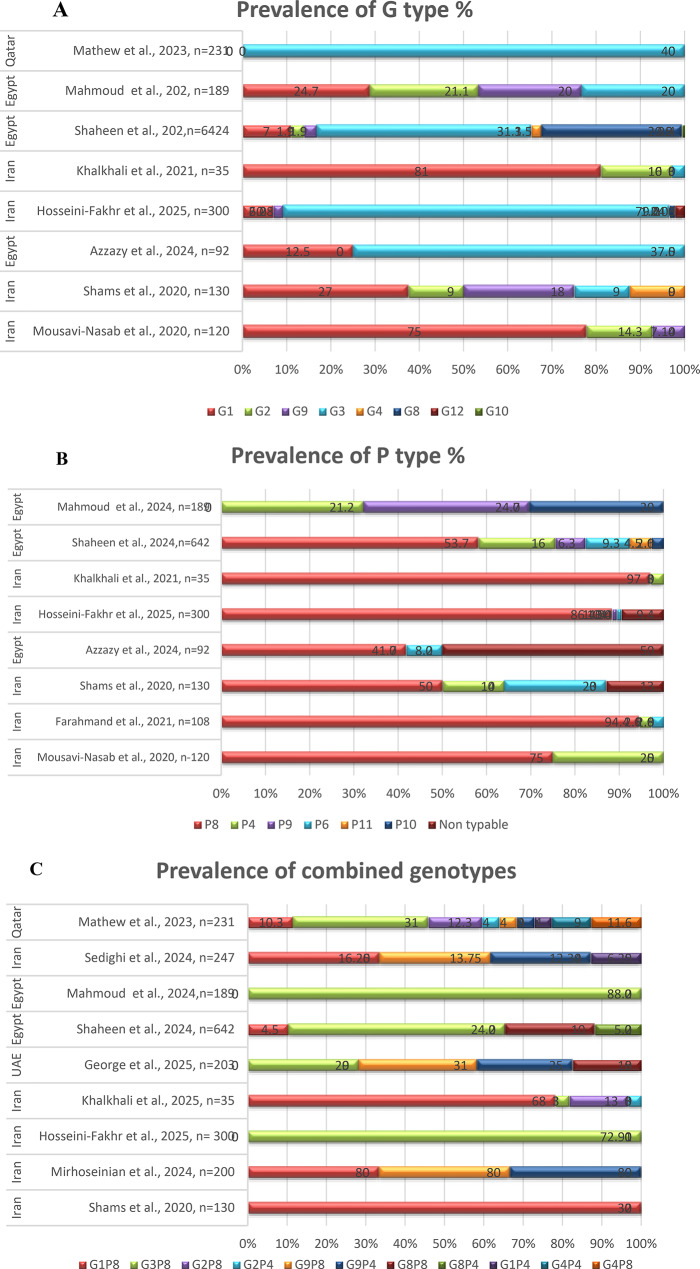
(**A–B**): Prevalence and distribution of Rotavirus genotypes among children under five years of age across 9 Middle Eastern studies. Horizontal bar plots illustrate the prevalence of individual **G** genotypes (G1–G12) and combined G/P genotypes (e.g., G1P[8], G2P[4]); colored segments represent the proportion of each genotype. (**C)**: Prevalence of combined genotypes. The comparative view highlights regional variations, dominance patterns, and the occurrence of both mono-genotype and mixed genotype combinations

A total of 43 studies, encompassing 22,021 children from nine countries, were included in the analysis. Rotavirus was the predominant pathogen, reported in 65.1% (28/43) of studies, with 18 studies performing genotyping Figs. 4A–C. The most frequent G genotypes were G1, G2, G3, and G9. Genotype G3 showed particularly high prevalence in Iran (79.2%) and Qatar (40%), while G1 varied geographically from 24.7% in Egypt to 81% in Iran. Less common genotypes such as G8, G10, and G12 were reported sporadically. Among P genotypes, P[8] predominated, followed by P[4] and P[6], with a notable proportion of non-typable P strains (up to 50%) in several Egyptian studies. The most common G/P combinations were G3P[8] and G1P[8], with additional strains such as G2P[4], G9P[8], and reassortant variants (e.g., G8P[4] and G4P[8]) reflecting ongoing viral genetic diversity and evolution in the region (Table 2). Other enteric viruses were detected less frequently. Adenovirus, predominantly type 41, was reported in 30.2% (13/43) of studies, often in co-infections. Norovirus appeared in 27.9% (12/43), with genotyping identifying GII.4 and recombinant GII.4[P16]. Astrovirus was reported in 23.3% (10/43), mainly type 1, with occasional MLB2 detection. Human bocavirus and sapovirus each appeared in 6.98% (3/43) of studies, while enterovirus, bocaparvovirus, coxsackievirus A16, influenza A (H3), RSV-A, and parechovirus were rare (2.33% each), typically as incidental findings.

**Table 1:** Characteristics of included studies investigating viral etiology of acute gastroenteritis in children in the Middle East 2020–2025

Study ID	Study Author	Year	Country	Virus Detected	Study Design	Sample Size	Diagnostic Method	Risk of Bias
ST01 [9]	Mousavi-Nasab et al.	2020	Iran	Rotavirus	Cross-sectional	120	RT-PCR	Moderate
ST02 [10]	Mohammad et al.	2020	Kuwait	Human Adenovirus, Norovirus GII Enteroviruses +, Astrovirus MLB2, Bocaparvovirus1	Cross-sectional	84	Metagenomics + Multiplex RT-PCR	Moderate
ST03 [11]	Farahmand et al.	2021	Iran	Rotavirus genotypes	Cross-sectional	108	Sanger sequencing	Low
ST04 [12]	El-Senousy	2020	Egypt	Rotavirus	Cross-sectional	1026	Nested RT-PCR	Low
ST05 [13]	Abdel-Rahman et al.	2021	Qatar	Rotavirus, Norovirus, Adenovirus	Cross-sectional	736	FilmArray GI Panel	Low
ST06 [14]	Fallah et al.	2024	Iran	Rotavirus variants	Cross-sectional	187	ELISA RT-PCR + sequencing	Moderate
ST07 [15]	Iflah et al.	2021	Israel	Various AGE causes	Case-control	3573	ELISA + RT-PCR	Low
ST08 [16]	Shams et al.	2020	Iran	Rotavirus G/P types	Cross-sectional	130	RT-PCR	Moderate
ST09 [17]	Rajabnejad et al.	2024	Iran	Rotavirus, Norovirus, Adenovirus, Sapovirus, Astrovirus, SARS-CoV-2	Cross-sectional	84	Multiplex RT-PCR	Moderate
ST010 [18]	Mathew et al.	2021	Qatar	Rotavirus	Cross-sectional	687	RT-PCR+ sequencing	Low
ST011 [19]	Alsuwaidi et al.	2021	Emirates	Rotavirus, Norovirus GII, Adenovirus	Case–control	276	Multiplex PCR	Moderate
ST012 [20]	Rizk et al.	2021	Egypt	Human Bocavirus, Rotavirus, Norovirus, Adenovirus, Astrovirus	Cross-sectional	102	RT-PCR	Moderate
ST013 [21]	Yasaie et al.	2024	Iran	Rotavirus, Norovirus, Astrovirus, Enterovirus	Cross-sectional	130	RT-PCR	Moderate
ST014 [22]	Omar et al.	2024	Israel	Rotavirus	Retrospective study	1419	RT-PCR	Low
ST015 [23]	Azzazy et al.	2024	Egypt	Rotavirus	Cross-sectional	92	ELISA + RT-PCR	Moderate
ST016 [24]	Mishra et al.	2020	Lebanon	Rotavirus	Genomic surveillance	428	Whole genome sequencing	Moderate
ST017 [25]	Mirhoseinian et al.	2024	Iran	Rotavirus	Genomic surveillance	200	Whole genome sequencing	Moderate
ST018 [26]	Latifi et al.	2022	Iran	Rotavirus	Retrospective	82	PCR + sequencing	Moderate
ST019 [27]	Hosseini-Fakhr et al.	2025	Iran	Rotavirus	Cross-sectional	300	Genome sequencing	Low
ST020 [28]	Harastani et al.	2020	Lebanon	Rotavirus	Prospective	132	PCR + sequencing	Moderate
ST021 [29]	Khalkhali et al.	2021	Iran	Rotavirus	Molecular genetic (analytical)	35	Sequencing	Moderate
ST022 [30]	Mohammadi M et al.	2020	Iran	Bocavirus, Rotavirus, RSV	Cross-section	500	PCR / RT-PCR	Moderate
ST023 [31]	George et al.	2024	UAE	Rotavirus	Cross-section	203	Nanopore sequencing	Moderate
ST024 [32]	Danino et al.	2023	Israel	Rotavirus, Norovirus, Adenovirus (40/41), Astrovirus, Sapovirus	Prospective multicenter	5879	Multiplex PCR	Low
ST025 [33]	Ayyed et al.	2020	Iraq	Rotavirus	Cross-sectional	600	ELISA / PCR	Moderate
ST026 [34]	Montasser et al.	2022	Egypt	Adenovirus, Rotavirus	Cross-sectional	150	PCR	Moderate
ST027 [35]	Salavatiha et al.	2024	Iran	Norovirus, Rotavirus, Human Bocavirus, Adenovirus	Cross-sectional	100	PCR / RT-PCR	High
ST028 [36]	Allayeh et al.	2022	Egypt	Adenovirus	Longitudinal observational	447	PCR	Low
ST029 [37]	Kachooei et al.	2024	Iran	Astrovirus, Rotavirus	Cross-sectional	200	PCR+sequencing	Moderate
ST030 [38]	Kachooei et al.	2023	Iran	Rotavirus	Molecular genetic (analytical)	48	RT-PCR + sequencing	High
ST031 [39]	Shaheen et al.	2024	Egypt	Rotavirus	Cross-sectional	642	Semi-nested multiplex RT-PCR, immunochromatographic assay	Low
ST032 [40]	Zaraket et al.	2020	Lebanon	Rotavirus, Adenovirus	Retrospective	308	Immunoassay rapid test	Moderate
ST033 [41]	Khalife et al.	2025	Lebanon	Rotavirus, Adenovirus	Cross-sectional	400	Immunoassay rapid test	Low
ST034 [42]	Alqurayn et al.	2024	Saudi Arabia	Rotavirus, Adenovirus	Retrospective	478	Immunoassay rapid test	Low
ST035 [43]	Mahmoud et al.	2024	Egypt	Rotavirus	Cross-sectional	189	ELISA followed by nested PCR	Low
ST036 [44]	Sedighi et al.	2024	Iran	Rotavirus	Case series	284	ELISA + PCR	Moderate
ST037 [45]	Eftekhari et al.	2023	Iran	Norovirus	Cross-sectional	200	PCR+Sequencing + RT-PCR	Moderate
ST038 [46]	Othma et al.	2022	Egypt	Rotavirus، Norovirus Astrovirus، Adenovirus	Cross-sectional	50	Multiplex PCR	Moderate
ST039 [47]	Mathew et al.	2023	Qatar	Rotavirus	Cross-sectional	231	PCR+Sequencing	Moderate
ST040 [48]	Alsubaiei et al.	2023	Saudi Arabia	Rotavirus, Norovirus GI, Norovirus GII, Astrovirus, Enteric Adenovirus, Enterovirus	Cross-sectional	92	PCR /RT-PCR	Moderate
ST041 [49]	Hijazi et al.	2022	Qatar	Adenovirus، Coxsackievirus A16، Rotavirus, ، Norovirus، Influenza A virus، Respiratory syncytial virus	Cross-sectional	89	Metagenomics Sequencing	Moderate
ST042 [50]	Mashaly et al.	2022	Egypt	Human parechovirus، Rotavirus، Norovirus، Astrovirus	Cross-sectional	100	RT-PCR, ELISA, rapid Immunoassay	Moderate
ST043 [51]	Motamedi-Rad et al.	2020	Iran	Rotavirus	Cross-sectional	361	PCR	Moderate

**Table 2:** Prevalence, genotypic variation, and geographic distribution of viruses in the included studies

Virus Type	Genotypes detected	Prevalence (% Among studies (*n* = 43))	Countries Reported	Key Findings
Rotavirus	G1, G2, G3, G4, G8, G9, G10, G12; P[4], P[6], P[8], P[9], P[10], P[11]; G1P[8], G2P[4], G3P[8], G4P[8], G9P[8], G8P[4], G2P[8], G9P[4], G8P[8], G1P[4], G4P[4].	65.1% (28/43)	Iran, Egypt, Qatar, Lebanon, UAE, Israel, Iraq, Saudi Arabia and Kuwait	Most common virus. Out of 28, 18 molecular studies detected Rotavirus genotypes. G3P[8] and G1P[8] were the most prevalent. Mixed and reassortant types reported.
Norovirus	GII.4, GII.3, GII.7, GII.8, GII.17; GII.P4, P7, P8, P16; GII.4[P16] recombinant	27.9% (12/43)	Iran, Egypt, Qatar, UAE, Israel, Saudi Arabia, Kuwait	Only 1 molecular study differentiated the genotypes. Norovirus GII.4 was the most dominant—frequent co-infection and recombinants like GII.4[P16].
Adenovirus	Types 41 (enteric), 40, 1, 6	30.2% (13/43)	Egypt, UAE, Qatar, Iran, Lebanon, Israel, Saudi Arabia, Kuwait	Only 1 molecular study detected the genotype. Adenovirus 41 was the main type in GE. Often found in co-infection.
Astrovirus	HAstV-1 to -8 (mainly type 1), MLB2	23.3% (10/43)	Iran, Egypt, Qatar, Israel, Saudi Arabia, Kuwait, UAE	Limited molecular typing. Astrovirus detected at low frequency.
Bocavirus	HBoV-1, HBoV-2, HBoV-3, HBoV-4 (often reported as HBoV-2/4)	6.9% (3/43)	Egypt, Iran	Genotyping has been detected in only 1 molecular study. Frequently appeared in mixed infections with RVA or ADV.
Sapovirus	Genogroups GI, GII (often untyped genotypes)	6.9% (3/43)	UAE, Qatar, Israel	Detected infrequently, often without detailed genotyping.
Enterovirus	Not always genotyped	6.9% (3/43)	Kuwait, Iran, Saudi Arabia	Occasionally detected by PCR, sometimes in co-infection.
Bocaparvovirus	Not specified	2.3% (1/43)	Kuwait	Rarely detected. Mostly from advanced molecular testing.
Coxsackievirus	A16	2.3% (1/43)	Qatar	Identified as part of a co-infection panel.
Influenza A (H3)	H3 subtype	2.3% (1/43)	Qatar	Detected only in multiplex panels.
RSV A	Subtype A	2.3% (1/43)	Qatar	Occasionally detected in stool by PCR.
Parechovirus	HPeV (not always typed)	2.3% (1/43)	Egypt	Reported as an incidental finding.

### Pooled prevalence analysis

**Fig. 5:**
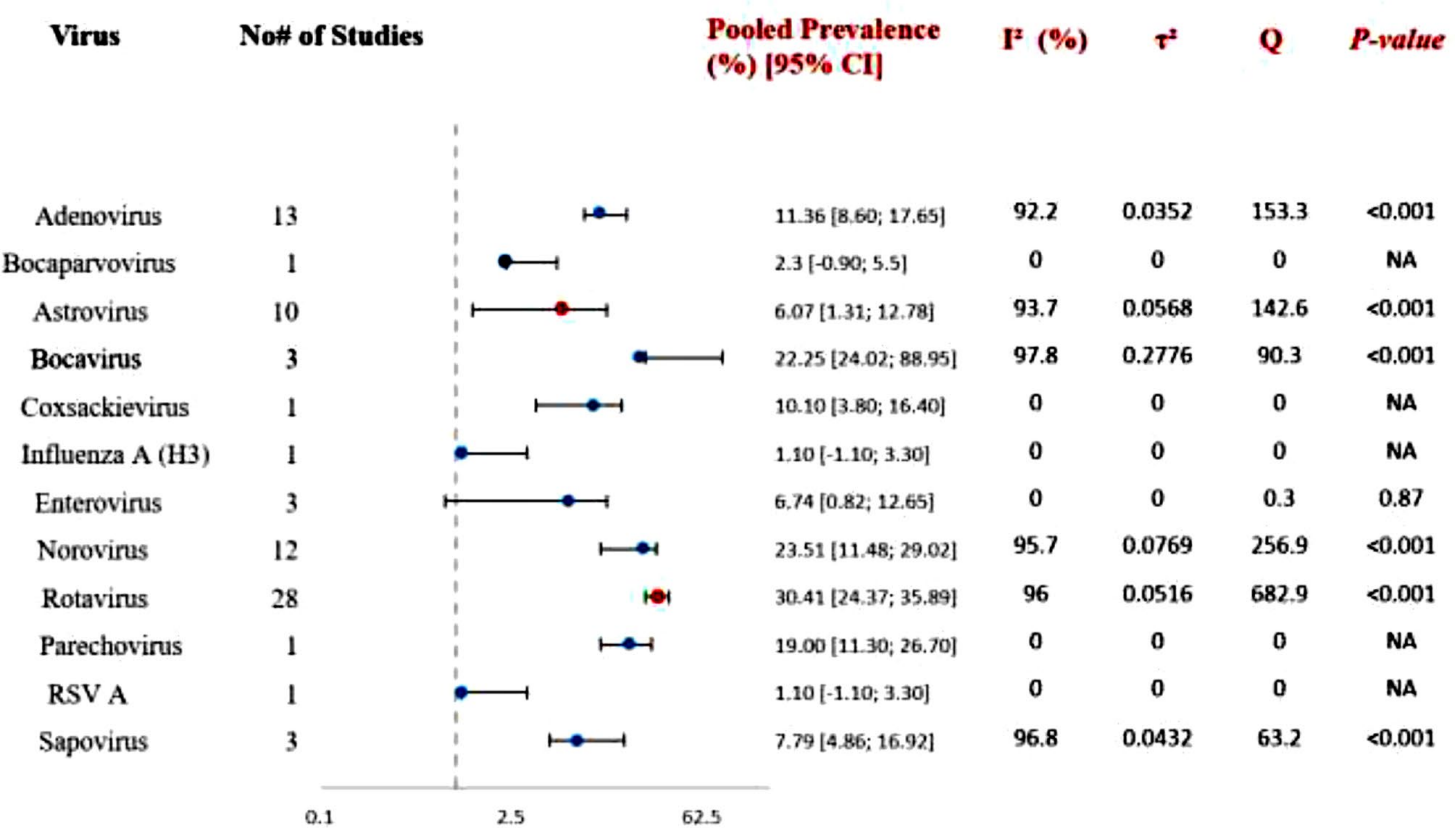
Forest plot of pooled prevalence estimates of enteric viruses among children under five years in the Middle East (2020–2025). The plot presents pooled prevalence estimates derived using a random-effects model (DerSimonian–Laird) with Freeman–Tukey transformation with corresponding 95% confidence intervals (CI) and the number of contributing studies (k) for each virus. I² represents the percentage of variability attributed to heterogeneity, τ² indicates the between-study variance, and Cochran’s Q with its p-value evaluates statistical heterogeneity. Values reported as 0 occur when the estimate is derived from a single study (k = 1). NA: Not applicable

The pooled prevalence estimates of viral pathogens associated with gastroenteritis among children under five years of age in the Middle East are summarized in Fig. 5. Rotavirus demonstrated the highest pooled prevalence at 30.4% (95% CI: 24.3–35.8) based on 28 studies, followed by norovirus (23.5%, 95% CI: 11.4–29.0; 12 studies). Adenovirus was detected with a pooled prevalence of 11.3% (95% CI: 8.6–17.6; 13 studies), while astrovirus showed a lower prevalence of 6.0% (95% CI: 1.3–12.7; 10 studies). Sapovirus was identified in a smaller number of studies with a pooled prevalence of 7.7% (95% CI: 4.8–16.9; 3 studies). Bocavirus showed a relatively high pooled estimate (22.2%), although this result was derived from only three studies and was associated with a wide confidence interval, indicating substantial uncertainty. Other viruses, such as parechovirus, enterovirus, coxsackievirus, influenza A (H3), and RSV A, were reported in only one or a small number of studies, limiting the ability to generate reliable pooled estimates. Across the major viruses, very high heterogeneity was observed. Rotavirus exhibited an I² of 96%, norovirus 95.7%, astrovirus 93.7%, adenovirus 92.2%, and sapovirus 96.8%, indicating considerable variability in prevalence estimates between studies. The Cochran’s Q test was statistically significant (*p* < 0.001) for most viruses, confirming the presence of significant between-study heterogeneity. In contrast, heterogeneity could not be meaningfully evaluated for viruses represented by only a single study.

**Fig. 6:**
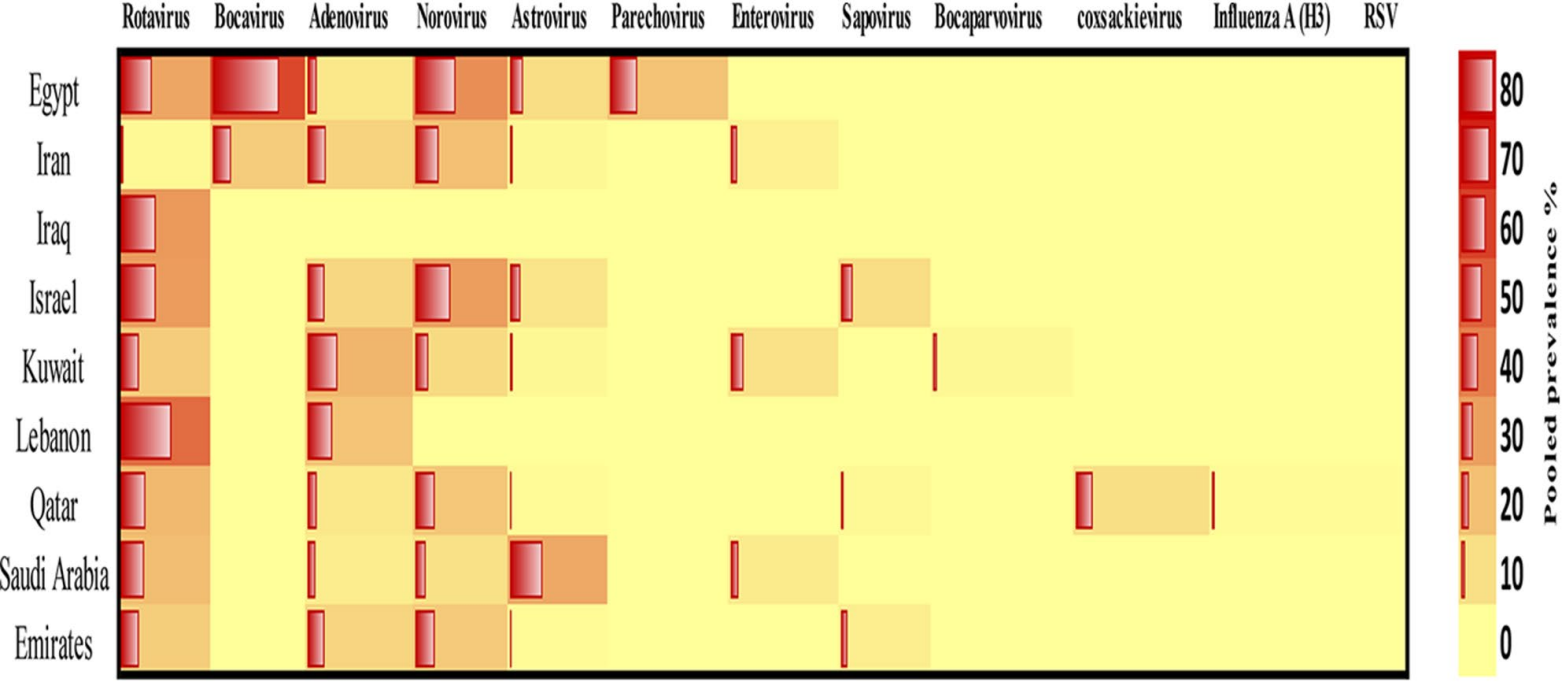
Heatmap of the geographical distribution and pooled prevalence of key viral pathogens across nine Middle Eastern countries. Rows represent individual countries, and columns represent the investigated viral pathogens. The color gradient (yellow to dark red) reflects increasing prevalence values, with darker shades indicating higher rates. Minimally colored cells denote absence or very low prevalence

A heatmap showing the distribution and relative intensity of viral detection across the included studies is presented in Fig. 6. The color intensity reflects the pooled prevalence of each virus across the analyzed studies. Darker color intensities indicate higher pooled prevalence values, whereas lighter shades represent lower prevalence estimates. The heatmap demonstrates a heterogeneous pattern, highlighting noticeable variability in the pooled viral prevalence reported across different studies. Several studies also reported the detection of multiple viruses within the same study population.

### Co-infection patterns across the included studies

After a detailed review of the included studies, twelve studies were included in the analysis of co-infections. Infection patterns were summarized descriptively rather than pooled quantitatively due to the inconsistency in denominator definitions across studies. The detailed prevalence estimates for the different infection patterns are presented in Table 3, and the reported percentages were kept as originally published by each study without recalculation. The pattern of mixed viral infections was predominantly observed among major enteric viruses, particularly combinations involving Rotavirus, Norovirus, and Adenovirus, which constituted the most frequently reported co-infection clusters across studies conducted in Egypt, Saudi Arabia, Lebanon, and other Middle Eastern countries. Dual infection involving Rotavirus A and Norovirus was particularly highlighted in Othman et al. 2022 from Egypt and Alsubaiei et al. 2023 from Saudi Arabia, where mixed viral detection represented a noticeable proportion of positive samples. Similarly, Rotavirus combined with Adenovirus was reported in Egyptian and Lebanese studies, including Montasser et al. 2022, Zaraket et al. 2020, and Khalife et al. 2025, although the reported frequencies varied across datasets. Triple viral infections were relatively uncommon and were mainly observed in clusters involving Rotavirus, Norovirus, and Adenovirus or Rotavirus, Norovirus, and Astrovirus, as documented in Othman et al. 2022. In addition, infections involving Human Bocavirus and Human Parechovirus were detected at lower and more variable rates, mostly in association with Rotavirus or Adenovirus, as reported in studies from Egypt, including Rizk et al. 2021 and Mashaly et al. 2022. Overall, the distribution of co-infection patterns was heterogeneous across the included studies, reflecting variability in population characteristics, diagnostic techniques, and study designs.

**Table 3:** Summarized distribution of mono-infection and co-infection patterns of enteric viruses among included studies

Study ID	Study (First Author, Year)	Sample Size	Virus Type(s) Investigated	Infection Pattern and Main Findings
ST02	Mohammad et al., 2020 [10]	84	Human Adenovirus, Norovirus GII Enteroviruses +, Astrovirus MLB2, Bocaparvovirus1	Co-infection: Human Adenovirus with other viruses (9; 20.9%); Bocavirus-1 + Adenovirus (1; 2.3%); Astrovirus MLB2 + Human Enterovirus + Enterovirus A (1; 2.3%).
ST05	Abdel-Rahman et al., 2020 [13]	736	Rotavirus, Norovirus, Adenovirus	Mono-infection: 351 (47.7%); Co-infection: 36 (4.9%).
ST09	Rajabnejad et al., 2024 [17]	84	SARS-CoV-2, Norovirus, Sapovirus, Rotavirus, Adenovirus, Astrovirus	Co-infection: Two viruses detected (23.6%); Three viruses detected (1.8%).
ST011	Alsuwaidi et al., 2021 [19]	276	Adenovirus, Astrovirus, Norovirus GI/GII, Rotavirus, Sapovirus	Mono-infection: 104 (37.7%); Co-infection: 77 (27.9%).
ST012	Rizk et al., 2021 [20]	102	Human Bocavirus, Rotavirus, Norovirus, Adenovirus, Astrovirus	Mono-infection: 52.5% (31/59); Co-infection: Bocavirus + Adenovirus 1.7% (1/59); Bocavirus + Rotavirus 33.9% (20/59); Bocavirus + Rotavirus + Adenovirus 11.9% (7/59).
ST022	Mohammadi M et al., 2020 [30]	500	Rotavirus, Bocavirus	Co-detection: Rotavirus + Bocavirus (62.5%).
ST024	Danino et al., 2023 [32]	5,879	Rotavirus, Norovirus, Adenovirus (40/41), Astrovirus, Sapovirus	Mono-infection: 2,662 (45.3%); Co-infection: 245 (9.2%).
ST026	Montasser et al., 2022 [34]	150	Adenovirus, Rotavirus	Co-infection: Rotavirus + Adenovirus (8%; 12/150).
ST032	Zaraket et al., 2020 [40]	280	Rotavirus, Adenovirus	Co-infection: 26 (9.3%).
ST033	Khalife et al., 2025 [41]	400	Rotavirus, Adenovirus	Co-infection: 22/400 (5.5%).
ST038	Othman et al., 2022 [46]	50	Norovirus GII, Rotavirus, Astrovirus, Adenovirus	**Two-virus co-infection**: 18% (9/50) including Rotavirus A + Norovirus GII 12% (6/50), Norovirus GII + Astrovirus 4% (2/50), Norovirus GII + Adenovirus 2% (1/50); **Three-virus co-infection**: 4% (2/50) including Rotavirus A+ Norovirus GII+ Adenovirus 2% (1/50), and Rotavirus A+ Norovirus GII+ Astrovirus 2% (1/50).
ST040	Alsubaiei et al., 2023 [48]	92	Rotavirus, Norovirus GI, Norovirus GII, Astrovirus, Enteric Adenovirus, Enterovirus	Mono-infection prevalence: 79.3% (50/63) and Coinfection 20.6%(13/63).
ST042	Mashaly et al., 2022 [50]	100	Human parechovirus، Rotavirus، Norovirus، Astrovirus	Mono-infection: 5%; Co-infection with Rotavirus (43.9%), Norovirus (36.8%), and Rotavirus + Norovirus (15.8%).

### Risk of bias analysis

Among the assessed studies, 65.11% were assessed as having a moderate risk of bias, 30.2% showed a low risk, and only 4.7% were classified as high-risk (Table 4). The predominance of low- to moderate-risk studies reflects an overall acceptable methodological quality, supporting their inclusion in the systematic review. Most studies demonstrated sufficient rigor in sample selection, data collection, and statistical analysis. However, the most common limitation observed across studies was the inadequate consideration or control of confounding factors. Examples include age, nutritional status, vaccination history, socioeconomic conditions, and co-infections with other enteric pathogens, all of which could influence the observed prevalence and severity of viral gastroenteritis and contribute to the moderate risk ratings in several studies. Importantly, all studies met the predefined eligibility criteria, including target population, geographic location, study period, and viral etiology focus. These findings confirm the suitability and reliability of the included studies for evidence synthesis and critical appraisal (Supplementary Table 1).

The results of the sensitivity analysis (Table 5) demonstrated that, excluding studies assessed as high risk of bias, the pooled prevalence estimates for most viruses showed minimal variation, with substantial overlap in 95% confidence intervals, indicating numerical stability of the meta-analytic results. Adenovirus, Astrovirus, Enterovirus, Norovirus, and Rotavirus demonstrated largely unchanged point estimates following sensitivity analysis.

**Table 4:** Overall risk of bias distribution

Overall Risk of Bias	Number of Studies (43)	Percentage (%)
Low (L)	13	30.2%
Moderate (M)	28	65.1%
High (H)	2	4.7%

**Table 5:** Sensitivity analysis of pooled prevalence estimates after exclusion of high-risk studies

Virus	Original pooled prevalence % (95% CI)	Sensitivity pooled prevalence % (95% CI)	k (remaining studies)	Interpretation
Adenovirus	11.3 (8.6–17.6)	11.4 (8.0–15.4)	12	Robust
Astrovirus	6.0 (1.3–12.7)	6.0 (1.3–12.7)	10	Robust
Bocaparvovirus	2.3 (− 0.9–5.5)	2.3 (− 0.9–5.5)	1	Single-study
Bocavirus	22.2 (24.0–88.9)	36.2 (2.4–79.7)	2	Unstable
Coxsackievirus	10.1 (3.8–16.4)	10.1 (3.8–16.4)	1	Single-study
Influenza A (H3)	1.1 (− 1.1–3.3)	1.1 (− 1.1–3.3)	1	Single-study
Enterovirus	6.7 (0.8–12.6)	6.7 (0.8–12.6)	3	Robust
Norovirus	23.5 (11.4–29.0)	23.5 (8.6–29.6)	10	Stable
Rotavirus	30.4 (24.3–35.8)	30.4 (24.2–36.2)	27	Robust
Parechovirus	19.0 (11.3–26.7)	19.0 (11.3–26.7)	1	Single-study
RSV A	1.1 (− 1.1–3.3)	1.1 (− 1.1–3.3)	1	Single-study
Sapovirus	7.7 (4.8–16.9)	7.7 (4.8–16.9)	3	Robust

**k** indicates the number of studies included in the sensitivity analysis after exclusion of studies assessed as high risk of bias

**Robust** indicates minimal change in the pooled estimates after excluding high-risk studies

**Stable** indicates similar pooled estimates with minor changes in confidence intervals

**Unstable** indicates large changes or wide confidence intervals due to a small number of studies

**Single-study** indicates that only one study was available, and sensitivity analysis was not applicable

Rotavirus maintained the highest pooled prevalence after sensitivity testing (30.4%, 95% CI: 24.2–36.2), showing negligible deviation from the original estimate (30.4%). Similarly, Norovirus remained consistent, with prevalence estimates of 23.5% (95% CI: 8.6–29.6) compared to the original estimate of 23.5% (95% CI: 11.4–29.0). Adenovirus also exhibited only a minor change (11.3% to 11.4%), while Astrovirus (6.0%) and Enterovirus (6.7%) showed identical or near-identical prevalence estimates before and after exclusion. For viruses represented by a single study (Bocaparvovirus, Coxsackievirus, Influenza A (H3), Parechovirus, and RSV A), the sensitivity analysis was not applicable. Bocavirus demonstrated greater sensitivity to study exclusion, with the pooled prevalence increasing from 22.2% to 36.2%, and confidence intervals remained wide in both analyses, likely due to the inclusion of only two studies, which limited the precision of the estimate. Overall, the sensitivity analysis supports the general stability of pooled prevalence estimates for most viral pathogens, although caution is warranted when interpreting results derived from a small number of studies.

## Discussion

This systematic review and meta-analysis synthesized evidence from 43 studies conducted in nine Middle Eastern countries between 2020 and 2025, examining the epidemiology and genetic diversity of viral gastroenteritis among children under five years of age during the post-COVID-19 period. Although the review provides a broad regional overview, the included studies represent only nine of the sixteen countries in the Middle East. Moreover, more than half of the studies originated from Iran and Egypt, while several countries, including Yemen, Bahrain, Oman, Syria, and Palestine, lacked eligible data. This uneven geographic representation likely reflects differences in surveillance capacity, laboratory infrastructure, research funding, and political or socioeconomic challenges that may limit systematic epidemiological monitoring and reporting across parts of the region [52]. Consequently, the pooled estimates should be interpreted with caution, as they may more strongly reflect epidemiological patterns in countries with higher research output rather than uniformly representing the entire region. The pooled prevalence estimates identified Rotavirus as the most frequently detected viral pathogen associated with pediatric gastroenteritis in the region, followed by norovirus, adenovirus, and astrovirus. These findings are broadly consistent with previous regional and global studies that have identified Rotavirus and norovirus as the leading viral causes of acute gastroenteritis in young children [1, 53, 54]. However, substantial heterogeneity was observed across studies, with consistently high I² values exceeding 90% for several major viruses. Such levels of heterogeneity indicate considerable variability in prevalence estimates between studies. This variability likely reflects differences in study design, sample size, healthcare settings, timing of specimen collection, and diagnostic methodologies rather than epidemiological variation alone. Geographic mapping also suggested differences in virus circulation patterns among countries, which may be influenced by variations in population density, sanitation conditions, healthcare access, and the implementation of vaccination programs [55–57]. Genotypic analysis of Rotavirus demonstrated considerable genetic diversity across the region. The most frequently reported genotypes included G1, G2, G3, and G9, commonly detected in combination with P[8], P[4], and P[6] types. Among these, genotype constellations such as G3P[8] and G1P[8] were the most prevalent, while occasional reassortant or non-typable strains, including G8P[4], were also reported. These findings are consistent with previously published surveillance studies describing similar genotype distributions across Europe and the Eastern Mediterranean region [58]. The presence of diverse genotype combinations, including reassortant strains, highlights the genetic diversity of circulating rotavirus strains and underscores the importance of continuous molecular surveillance. Data on norovirus genotypes were more limited but generally aligned with global epidemiological patterns. The majority of reported strains belonged to genotype GII.4 and recombinant variants such as GII.4[P16], which are commonly associated with global outbreaks [59]. Adenovirus infections were primarily attributed to type 41, a well-recognized etiological agent of pediatric gastroenteritis [60], while astrovirus infections were most frequently associated with type 1 [61]. Overall, these findings indicate that the viral strains circulating in the Middle East largely mirror global genotype distributions. Co-infection with multiple enteric viruses was reported in a notable proportion of cases across the included studies. Mixed infections involving combinations of Rotavirus, adenovirus, norovirus, and bocavirus were frequently observed, while triple viral infections were less common and were reported mainly in studies conducted in Egypt [51]. The increasing recognition of viral co-infections may partly reflect the expanding use of advanced molecular diagnostic techniques, such as multiplex PCR panels and metagenomic sequencing, which enable simultaneous detection of multiple pathogens within a single sample [55, 56, 62]. Nevertheless, the clinical significance of viral co-infections remains uncertain, as the presence of multiple viruses does not necessarily imply that each pathogen contributes equally to disease severity. In addition, the relatively higher prevalence of bocavirus reported in some studies may reflect detection bias associated with the use of highly sensitive molecular assays. The COVID-19 pandemic represents an important contextual factor during the study period. Public health interventions implemented in many countries during 2020–2021, including lockdown measures, improved hygiene practices, and reduced healthcare utilization, may have influenced patterns of virus transmission and detection. Several studies reported changes in viral activity following the relaxation of pandemic restrictions, including increases in rotavirus and norovirus detection and shifts in circulating genotypes [63–66]. However, the observational nature of the included studies and the absence of standardized surveillance data limit the ability to draw causal conclusions regarding the impact of pandemic-related interventions. Sensitivity analyses excluding studies classified as having a high risk of bias produced minimal changes in the pooled prevalence estimates for most viruses. The persistence of similar point estimates and overlapping confidence intervals suggests that the main findings were not substantially influenced by study quality. An exception was observed for bocavirus, where the pooled estimate showed considerable instability following study exclusion, likely due to the small number of contributing studies and limited sample sizes [51, 67].

### Limitations and strengths

Several methodological limitations should be considered when interpreting the findings of this review. One important limitation relates to the inconsistency in the calculation of mono-infection and co-infection rates across the included studies. Some studies calculated these proportions relative to the total number of tested samples, whereas others reported percentages only among virus-positive cases. Because these denominators differ substantially, direct comparison or meta-analytic pooling of these rates could generate misleading estimates. To preserve data integrity and avoid inappropriate statistical aggregation, these values were therefore reported descriptively in a summary table exactly as presented in the original studies. Additional methodological variability further contributed to heterogeneity across studies. Diagnostic approaches ranged from conventional enzyme immunoassays and rapid antigen tests to advanced molecular techniques such as multiplex PCR and metagenomic sequencing. Differences in sensitivity and specificity across these platforms may have influenced the detection of certain viruses and the identification of co-infections, thereby affecting reported prevalence estimates. Furthermore, several viruses were represented by only a small number of studies, limiting the statistical power required for reliable quantitative analyses. The high heterogeneity observed in the present analysis highlights the challenges of synthesizing prevalence data across studies with diverse methodologies and contexts. Although the random-effects meta-analysis model accounts for some degree of between-study variability, the magnitude of heterogeneity suggests that pooled estimates should be interpreted as overall regional trends. Future research employing standardized diagnostic protocols, consistent reporting frameworks, and multicenter surveillance designs will be essential to reduce methodological variability and improve the comparability of epidemiological data across the region. A formal funnel plot analysis to assess publication bias was not performed. The combination of high methodological heterogeneity, differences in effect size definitions, and the limited number of studies for several viruses would make funnel plot interpretation unreliable and potentially misleading. Additional limitations include the exclusion of grey literature, such as conference abstracts and local surveillance reports, which may have led to underrepresentation of studies reporting non-significant findings or smaller sample sizes. Moreover, the lack of standardized reporting of rotavirus vaccination coverage across the included studies prevented evaluation of vaccine impact on viral prevalence and genotype distribution. Despite these limitations, the review has several important strengths. It includes a relatively large body of evidence from multiple countries during the post-COVID-19 period and integrates epidemiological findings with genotypic characterization of circulating viruses. The inclusion of studies employing advanced molecular diagnostic techniques enhanced the detection of viral pathogens and co-infections, providing a more comprehensive understanding of viral gastroenteritis epidemiology in the region. Collectively, these findings provide valuable insights into the epidemiology and genetic diversity of viral gastroenteritis among children under five years of age in 9 Middle Eastern countries, while also highlighting important gaps in surveillance and reporting that should be addressed in future research.

## Conclusion

This systematic review and meta-analysis confirm that Rotavirus and norovirus remain the predominant viral pathogens associated with pediatric gastroenteritis in the Middle East. The circulating viral populations demonstrate considerable genetic diversity, including multiple genotype constellations and occasional reassortant strains. Co-infections involving multiple enteric viruses were also frequently reported, particularly in studies employing advanced molecular diagnostic techniques. However, interpretation of the regional epidemiology is constrained by uneven geographic representation, substantial methodological heterogeneity, inconsistent reporting of infection patterns, and limited information on vaccination coverage. These factors highlight the need for strengthened molecular surveillance systems, standardized diagnostic and reporting protocols, and expanded multicenter studies across underrepresented countries. Such efforts will be essential to accurately monitor viral circulation, detect emerging strains, and support evidence-based public health interventions aimed at reducing the burden of viral gastroenteritis among children in the Middle East.

## Supplementary Information

Below is the link to the electronic supplementary material.

Supplementary Material 1

## Data Availability

All data generated or analyzed during this study are available in the submitted article and its supplementary information files.

## Abbreviations

AGE: Acute Gastroenteritis
RVA: Rotavirus A
PCR: Polymerase Chain Reaction
RT-PCR: Reverse Transcription PCR
VP: Viral Protein
G / P: Glycoprotein / Protease-sensitive protein
WHO: World Health Organization
MMR: Measles, Mumps, Rubella
CI: Confidence Interval
OR: Odds Ratio
RR: Relative Risk
IQR: Interquartile Range
COVID-19: Coronavirus Disease 2019
PRISMA: Preferred Reporting Items for Systematic Reviews and Meta-Analyses
RVA G/P: Rotavirus A genotypes
RNA: Ribonucleic Acid
DNA: Deoxyribonucleic Acid
HRV: Human Rotavirus
NV: Norovirus
HBoV: Human Bocavirus
AdV: Adenovirus
HAstV: Human Astrovirus
RSV: Respiratory Syncytial Virus
