# Supplementary material for: Epidemiology and genetic variation of acute viral gastroenteritis in children under five years in the Middle East (2020–2025): a systematic review and meta-analysis
Source: BMC Infect Dis. 2026 May 9;26:930. doi: 10.1186/s12879-026-13412-5 (PMC13162483; doi:10.1186/s12879-026-13412-5)
Supplement: Supplementary file 1 — Supplementary Material 1 [file 12879_2026_13412_MOESM1_ESM.docx]

**Supplementary Material**

1. **Detailed Search Strategies for All Databases**

A systematic and comprehensive literature search was performed to identify studies investigating the **epidemiology, prevalence, and genetic diversity of enteric viruses causing acute gastroenteritis** in children under five years of age in the Middle East. The search included studies published from **1 January 2020 to 31 May 2025**.

The following electronic databases were searched: **PubMed/MEDLINE, Scopus, Web of Science, ScienceDirect, and WHO Global Index Medicus**.

The search combined **controlled vocabulary** (e.g., MeSH in PubMed) with **free-text terms** related to:

- Disease condition (gastroenteritis, viral diarrhea)
- Viral pathogens (rotavirus, norovirus, adenovirus, astrovirus, sapovirus)
- Molecular characterization (genotyping, phylogenetic analysis, sequence analysis)
- Epidemiological outcomes (prevalence, incidence, distribution, burden)
- Target population (children under five, infants, preschool children)
- Geographic region (Middle East countries)

Boolean operators (**AND, OR**) were used to combine these concepts. The search was tailored to each database to optimize retrieval.

The Middle Eastern countries included in the search were: **Saudi Arabia, United Arab Emirates, Qatar, Kuwait, Bahrain, Oman, Yemen, Iraq, Syria, Lebanon, Jordan, Palestine, Egypt, Turkey, Iran, and Israel**.

The complete search strategies for each database are detailed below.

**1. PubMed / MEDLINE**

("gastroenteritis"[MeSH Terms] OR "acute viral gastroenteritis" OR "viral diarrhea" OR "diarrhea"[MeSH Terms])
AND
("rotavirus"[MeSH Terms] OR "norovirus" OR "adenovirus" OR "astrovirus" OR "sapovirus")
AND
("genotype" OR "genotyping" OR "genetic variation" OR "phylogenetic" OR "sequence analysis" OR "molecular epidemiology")
AND
("epidemiology"[MeSH Terms] OR "incidence" OR "prevalence" OR "distribution" OR "burden")
AND
("infant"[MeSH Terms] OR "child, preschool"[MeSH Terms] OR "under-five" OR "children under 5" OR "0–59 months")
AND
("Middle East" OR Afghanistan OR Bahrain OR Egypt OR Iran OR Iraq OR Israel OR Jordan OR Kuwait OR Lebanon OR Oman OR Palestine OR Qatar OR Saudi Arabia OR Syria OR Turkey OR "United Arab Emirates" OR UAE OR Yemen)

**Filters applied:**

- Publication date: 1 January 2020 – 31 May 2025
- Species: Humans
- Age group: Birth–5 years
- Language: English, Arabic

**2. Scopus**

TITLE-ABS-KEY("acute viral gastroenteritis" OR "viral diarrhea" OR "gastroenteritis")
AND
TITLE-ABS-KEY("rotavirus" OR "norovirus" OR "adenovirus" OR "astrovirus" OR "sapovirus")
AND
TITLE-ABS-KEY("genotype" OR "genotyping" OR "genetic variation" OR "phylogenetic" OR "sequence analysis" OR "molecular epidemiology")
AND
TITLE-ABS-KEY("epidemiology" OR "incidence" OR "prevalence" OR "distribution" OR "burden")
AND
TITLE-ABS-KEY("child*" OR "infant" OR "under-five" OR "0-59 months")
AND
TITLE-ABS-KEY("Middle East" OR Bahrain OR Egypt OR Iran OR Iraq OR Israel OR Jordan OR Kuwait OR Lebanon OR Oman OR Palestine OR Qatar OR Saudi Arabia OR Syria OR Turkey OR "United Arab Emirates" OR UAE OR Yemen)

**Filters applied:**

- Publication years: 2020–2025
- Language: English, Arabic
- Document type: Article
- Subject area: Medicine, Microbiology, Infectious Diseases

**3. Web of Science**

TS=("acute viral gastroenteritis" OR "viral diarrhea" OR "gastroenteritis")
AND
TS=("rotavirus" OR "norovirus" OR "adenovirus" OR "astrovirus" OR "sapovirus")
AND
TS=("genotype" OR "genotyping" OR "genetic variation" OR "phylogenetic" OR "sequence analysis" OR "molecular epidemiology")
AND
TS=("epidemiology" OR "incidence" OR "prevalence" OR "distribution" OR "burden")
AND
TS=("child*" OR "infant" OR "under-five" OR "0–59 months")
AND
TS=("Middle East" OR Bahrain OR Egypt OR Iran OR Iraq OR Israel OR Jordan OR Kuwait OR Lebanon OR Oman OR Palestine OR Qatar OR Saudi Arabia OR Syria OR Turkey OR "United Arab Emirates" OR UAE OR Yemen)

**Filters applied:**

- Publication years: 2020–2025
- Language: English, Arabic
- Document type: Articles

**4. ScienceDirect**

("acute viral gastroenteritis" OR "viral diarrhea" OR "gastroenteritis")
AND
("rotavirus" OR "norovirus" OR "adenovirus" OR "astrovirus" OR "sapovirus")
AND
("genotype" OR "genotyping" OR "genetic variation" OR "phylogenetic" OR "sequence analysis" OR "molecular epidemiology")
AND
("epidemiology" OR "incidence" OR "prevalence" OR "distribution" OR "burden")
AND
("child*" OR "infant" OR "under-five" OR "0–59 months")
AND
("Middle East" OR Bahrain OR Egypt OR Iran OR Iraq OR Israel OR Jordan OR Kuwait OR Lebanon OR Oman OR Palestine OR Qatar OR Saudi Arabia OR Syria OR Turkey OR "United Arab Emirates" OR UAE OR Yemen)

**Filters applied:**

- Publication years: 2020–2025
- Language: English, Arabic
- Document type: Research articles

**5. WHO Global Index Medicus**

("acute viral gastroenteritis" AND
(rotavirus OR norovirus OR adenovirus OR astrovirus OR sapovirus) AND
(epidemiology OR incidence OR prevalence) AND
(genotyping OR genetic variation OR sequence OR phylogenetic) AND
(child OR infant OR under-five) AND
(Middle East OR Bahrain OR Egypt OR Iran OR Iraq OR Israel OR Jordan OR Kuwait OR Lebanon OR Oman OR Palestine OR Qatar OR Saudi Arabia OR Syria OR Turkey OR "United Arab Emirates" OR UAE OR Yemen))

**Filters applied:**

- Publication years: 2020–2025
- Region: Eastern Mediterranean Region
- Language: English, Arabic

1. **Risk of Bias Assessment Section**

Risk of bias was independently assessed by two reviewers using the **Joanna Briggs Institute (JBI) Critical Appraisal Checklists**, selected according to each study design (cross-sectional, cohort, or case-control).

Nine key methodological domains were evaluated for each study:

1. **Sample representativeness** – assessed based on whether the study population reflected the target population.
2. **Adequacy of sample size** – evaluated according to reported sample size calculations or whether the sample was large enough to ensure statistical precision.
3. **Clarity of sampling method** – judged on the description of recruitment and selection procedures.
4. **Validity of measurement instruments** – considered adequate if validated diagnostic assays (e.g., PCR, ELISA) or standardized case definitions were used.
5. **Standardization of data collection procedures** – assessed by whether uniform protocols were applied across participants.
6. **Control of confounding variables** such as age differences and other relevant demographic or clinical characteristics
7. **Appropriateness of statistical analysis** – assessed based on correct use of statistical tests, reporting of confidence intervals, and application of adjustment methods when necessary.
8. **Adequacy of response rates / missing data management** – evaluated for potential bias due to non-response or incomplete outcome data.
9. **Clarity of reporting** – judged based on completeness and transparency of methodological and results description.

Each domain was rated as **“Yes”**, **“No”**, or **“Unclear”**. An **overall risk-of-bias classification** was derived using the following rule:

- **Low risk:** ≥70% of domains rated “Yes”
- **Moderate risk:** 50–69% of domains rated “Yes”
- **High risk:** <50% of domains rated “Yes”

Discrepancies between reviewers were resolved through discussion and consensus. Studies classified as high risk were excluded in sensitivity analyses to assess the robustness of the pooled estimates, while studies with low and moderate risk were retained. Overall, most included studies were judged as low to moderate risk of bias.

**Supplementary Table 1: Quality appraisal results for all included studies across nine methodological domains.**

SR: Sample representativeness
SSA: Sample size adequate
SMC: Sampling method is clear
VMT: Valid measurement tool
SDC: Standardized data collection
CFA: Confounding factors addressed
SAA: Statistical analysis appropriate
RRA: Response rate adequate
ORB: Overall Risk of Bias

| Study ID | Author, Year | SR | SSA | SMC | VMT | SDC | CFA | SAA | RRA | ORB |
| --- | --- | --- | --- | --- | --- | --- | --- | --- | --- | --- |
| ST01 | Mousavi-Nasab et al., 2020 | L | M | M | L | L | H | M | N/A | M |
| ST02 | Mohammad et al., 2020 | L | M | M | L | L | H | M | N/A | M |
| ST03 | Farahmand et al., 2021 | L | L | M | L | L | H | M | N/A | L |
| ST04 | El Senousy W.M. et al., 2020 | L | L | M | L | L | H | M | N/A | L |
| ST05 | Abdel-Rahman et al., 2020 | L | L | L | L | L | L | L | N/A | L |
| ST06 | T. Fallah et al., 2024 | M | M | L | L | L | H | L | N/A | M |
| ST07 | Moti Iflah et al., 2021 | L | L | L | L | L | L | L | L | L |
| ST08 | Shams S et al., 2020 | M | M | L | L | M | H | M | N/A | M |
| ST09 | Rajabnejad M et al., 2024 | M | M | M | L | M | H | M | N/A | M |
| ST010 | Mathew et al., 2021 | L | L | L | L | L | L | L | L | L |
| ST011 | Alsuwaidi et al., 2021 | M | M | L | L | M | N/A | M | N/A | M |
| ST012 | Rizk et al., 2021 | M | M | L | L | M | H | M | N/A | M |
| ST013 | S. Yasaie et al., 2024 | H | M | L | L | M | H | M | N/A | M |
| ST014 | M. Omar et al., 2024 | L | L | L | L | L | L | L | L | L |
| ST015 | Azzazy et al., 2024 | M | M | L | L | M | M | M | N/A | M |
| ST016 | Mishra et al., 2020 | L | H | L | L | L | H | M | N/A | M |
| ST017 | Mirhoseinian et al., 2024 | H | H | M | L | L | H | M | N/A | M |
| ST018 | Latifi et al., 2022 | M | L | M | L | L | H | L | N/A | M |
| ST019 | Hosseini-Fakhr et al., 2025 | L | L | L | L | L | M | L | N/A | L |
| ST020 | Harastani et al., 2020 | L | L | L | L | L | M | L | N/A | M |
| ST021 | Khalkhali et al., 2021 | M | M | M | L | L | H | M | N/A | M |
| ST022 | Mohammadi M et al., 2020 | L | L | M | L | M | M | L | N/A | M |
| ST023 | George J.A. et al., 2024 | L | M | M | L | L | M | M | N/A | M |
| ST024 | Danino D. et al., 2023 | L | L | L | L | L | L | L | L | L |
| ST025 | Ayyed M.A. et al., 2020 | L | L | L | L | L | L | L | L | M |
| ST026 | Montasser et al., 2022 | M | M | L | L | M | M | M | N/A | M |
| ST027 | Salavatiha et al., 2024 | M | H | M | L | M | H | M | N/A | H |
| ST028 | Allayeh et al., 2022 | L | L | M | L | L | M | M | N/A | L |
| ST029 | Kachooei et al., 2024 | M | H | M | L | M | H | M | N/A | H |
| ST030 | Kachooei et al., 2023 | M | M | H | L | L | H | M | N/A | M |
| ST031 | Shaheen et al., 2024 | L | L | M | L | L | M | M | N/A | L |
| ST032 | Zaraket et al., 2020 | L | L | M | L | L | M | M | N/A | M |
| ST033 | Khalife et al., 2025 | L | L | M | L | L | L | M | L | L |
| ST034 | Alqurayn et al., 2024 | L | L | M | L | L | L | M | L | L |
| ST035 | Mahmoud et al., 2024 | L | L | M | L | L | L | M | N/A | L |
| ST036 | Sedighi et al., 2024 | L | L | M | L | M | H | M | N/A | M |
| ST037 | Eftekhari et al., 2023 | L | L | M | L | M | H | M | N/A | M |
| ST038 | Othma et al., 2022 | L | L | L | M | L | L | H | N/A | M |
| ST039 | Mathew et al., 2023 | L | M | L | M | L | L | M | N/A | M |
| ST040 | Alsubaiei et al., 2023 | M | M | M | L | M | H | M | N/A | M |
| ST041 | Hijazi et al., 2022 | M | M | M | H | L | M | H | N/A | M |
| ST042 | Mashaly et al., 2022 | M | H | M | L | M | H | L | N/A | M |
| ST043 | Motamedi-Rad et al., 2020 | L | L | L | L | L | H | L | N/A | M |

**Supplementary Table 2. Characteristics and Detailed Findings of Included Studies on Acute Viral Gastroenteritis in Children Under Five Years in the Middle East (2020–2025)**

| Study ID | Authors (Year) | Country | Sample Size | Virus Type | Main Findings (Detailed Results) |
| --- | --- | --- | --- | --- | --- |
| ST01 | Mousavi-Nasab et al. (2020) | Iran | 120 | Rotavirus | Genotyping showed predominance of G1 (75%), followed by G2 (14.3%) and G9 (7.14%), with mixed G1/G2 infections (3.58%). VP4 typing revealed P[8] in 75% and P[4] in 25% of isolates. |
| ST02 | Mohammad et al. (2020) | Kuwait | 84 | Multiple enteric viruses | Human adenovirus was most frequent (23.2%), followed by Rotavirus A (16.2%), Norovirus GII (11.6%), and enteroviruses (9.3%). Astrovirus MLB2 and Bocaparvovirus-1 were each detected in 2.3%. Mixed viral infections occurred in 20.9% of samples. |
| ST03 | Farahmand et al. (2021) | Iran | 108 | Rotavirus | All strains belonged to VP4 genotype P[II], predominantly P[8] (94.4%), while P[4] and P[6] each represented 2.8% of detected strains. |
| ST04 | El-Senousy (2020) | Egypt | 1026 | Rotavirus A | Human Rotavirus group A detected with overall prevalence ranging approximately between 24–37%. P genotypes were identified in 89.2% of strains and G genotypes in 46.4%. |
| ST05 | Abdel-Rahman et al. (2020) | Qatar | 736 | Multiple viruses | Among gastroenteritis cases, Norovirus was detected in 19.2%, Rotavirus in 18.5%, Adenovirus in 6.4%, Sapovirus in 2.2%, and Astrovirus in 1.35%. Overall viral positivity reached 47.7%. |
| ST06 | Fallah et al. (2024) | Iran | 187 | Rotavirus | Rotavirus detected in 27.3% of children. Single infections accounted for 76.9%, mixed infections 20.5%, and untypeable strains 2.5%. |
| ST07 | Iflah et al. (2021) | Israel | 3573 | Rotavirus | Rotavirus prevalence was significantly lower in children presenting with convulsions (2%) compared with those without convulsions (30%). |
| ST08 | Shams et al. (2020) | Iran | 130 | Rotavirus | VP6 positivity detected in 16.9%. G genotypes included G1 (27%), G9 (18%), and G2/G3/G4 (each 9%). P genotypes were P[8] (50%), P[6] (23%), and P[4] (14%). Predominant combination was G1P[8] (32%). |
| ST09 | Rajabnejad et al. (2024) | Iran | 84 | Multiple viruses | Overall viral detection reached 54.8%. Rotavirus was most common (43.6%), followed by Norovirus and Adenovirus (23.6%), Sapovirus and Coronavirus (14.5%), and Astrovirus (7.3%). Coinfections occurred in 23.6%, with triple infections in 1.8%. |
| ST10 | Mathew et al. (2021) | Qatar | 687 | Rotavirus | Rotavirus prevalence was 27.8%. Higher infection rates occurred among children aged 1–3 years (59.7%). Seasonal increase observed in summer (42.8%). Dominant genotype combinations included G3P[8], G2P[8], G4P[8], and G1P[8]. |
| ST11 | Alsuwaidi et al. (2021) | UAE | 276 | Multiple viruses | Rotavirus detected in 15.9%, Norovirus GII in 14.9%, Adenovirus in 13.4%, Sapovirus in 5.4%, Norovirus GI in 1.8%, and Astrovirus in 1.4%. Mono-infections occurred in 37.7% and coinfections in 27.9%. |
| ST12 | Rizk et al. (2021) | Egypt | 102 | Bocavirus | Human Bocavirus detected in 58% of samples. Genotypes included HBoV-3 (44%), HBoV-2/4 (33%), and HBoV-1 (30%). Coinfection with Rotavirus occurred in 33.9% of positive cases. |
| ST13 | Yasaie et al. (2024) | Iran | 130 | Multiple viruses | Rotavirus detected in 6.9%, Norovirus in 5.4%, Enterovirus in 4.6%, and Astrovirus in 2.3%. All Norovirus isolates belonged to genogroup GII. |
| ST14 | Omar et al. (2024) | Israel | 2072 | Rotavirus & Norovirus | Rotavirus detected in 25% of tested samples, whereas Norovirus prevalence was 12.1%. |
| ST15 | Azzazy et al. (2024) | Egypt | 92 | Rotavirus | Rotavirus prevalence was 24% by ELISA and 26.1% by nested RT-PCR. Genotype G3 predominated (37.5%). P[8] was most common (41.7%). High proportion of untypeable strains observed. |
| ST16 | Mishra et al. (2020) | Lebanon | 428 | Rotavirus | Emergence of G3P[6] reassortant strains with DS-1/Wa-like genomic constellation suggesting zoonotic reassortment events involving human and animal rotaviruses. |
| ST17 | Mirhoseinian et al. (2024) | Iran | 200 | Rotavirus | Genotype combinations G9P[4], G9P[8], and G1P[8] accounted for 80% of detected strains, with G9P[4] alone representing 30%. |
| ST18 | Latifi et al. (2022) | Iran | 82 | Rotavirus | VP8* gene analysis showed most P[8] strains clustered within lineage III, while a minority belonged to lineage IV. |
| ST19 | Hosseini-Fakhr et al. (2025) | Iran | 300 | Rotavirus | Rotavirus prevalence reached 32%. G3 genotype dominated (79.2%), followed by G1 and G9. P[8] detected in 86.4%. Most frequent combination was G3P[8] (72.9%). |
| ST20 | Harastani et al. (2020) | Lebanon | 132 | Rotavirus | Breakthrough infections identified among vaccinated children. Multiple genotype diversity observed including G1P[8], G2P[4], G4P[8], and G9P[8], with antigenic substitutions relative to vaccine strains. |
| ST21 | Khalkhali et al. (2021) | Iran | 35 | Rotavirus | G1 genotype dominated (81%). P[8] detected in 97% of strains. Main genotype combination was G1P[8] (78%). |
| ST22 | Mohammadi et al. (2020) | Iran | 500 | Rotavirus & Bocavirus | Rotavirus prevalence was 33.8%, while Bocavirus was detected in 14.4% of cases. |
| ST23 | George et al. (2024) | UAE | 203 | Rotavirus | VP7 genotype G9 predominated (56%). Common combinations included G9P[8] (31%), G9P[4] (25%), and G3P[8] (29%). |
| ST24 | Danino et al. (2023) | Israel | 5879 | Multiple viruses | Among positive samples, Rotavirus accounted for 35.3%, Norovirus 33%, Adenovirus 12.7%, Astrovirus 8.3%, and Sapovirus 10.7%. |
| ST25 | Ayyed et al. (2020) | Iraq | 600 | Rotavirus | Rotavirus prevalence was 32%, with higher infection rates observed among non-vaccinated children. |
| ST26 | Montasser et al. (2022) | Egypt | 150 | Rotavirus & Adenovirus | Rotavirus was detected in 58% of samples, while Adenovirus prevalence ranged between 6.7–8%. |
| ST27 | Salavatiha et al. (2024) | Iran | 100 | Multiple viruses | Norovirus was most frequent (32%), followed by Rotavirus (29%), Bocavirus (25%), and Adenovirus (14%). |
| ST28 | Allayeh et al. (2022) | Egypt | 447 | Adenovirus | Adenovirus prevalence was 7.8%, predominantly type 41 (71.2%), followed by type 40 (17.2%). |
| ST29 | Kachooei et al. (2024) | Iran | 200 | Astrovirus & Rotavirus | Human Astrovirus lineage 1b was identified for the first time in Iran. Atypical reassortant Rotavirus G1P[8] strain detected. |
| ST30 | Kachooei et al. (2023) | Iran | 48 | Rotavirus | Multiple reassortant genotypes detected, mainly G9P[4] and G9P[8] with different genomic constellations. |
| ST31 | Shaheen et al. (2024) | Egypt | 642 | Rotavirus | Rotavirus prevalence was 41.7%. Dominant G genotype was G3 (31.3%) and dominant combination G3P[8] (24.2%). |
| ST32 | Zaraket et al. (2020) | Lebanon | 308 | Rotavirus & Adenovirus | Rotavirus was detected in 72.1% and Adenovirus in 27.5% of children under five years. |
| ST33 | Khalife et al. (2025) | Lebanon | 400 | Rotavirus & Adenovirus | Rotavirus prevalence was 28%, Adenovirus 12.3%, and coinfection 5.5%. |
| ST34 | Alqurayn et al. (2024) | Saudi Arabia | 478 | Rotavirus & Adenovirus | Rotavirus was detected in 14.2% with seasonal peaks; Adenovirus prevalence 2.7% with year-round circulation. |
| ST35 | Mahmoud et al. (2024) | Egypt | 189 | Rotavirus | Major G genotypes were G1, G2, G3, and G9. Predominant combinations included G3P[8] and G1P[4]. |
| ST36 | Sedighi et al. (2024) | Iran | 247 | Rotavirus | Rotavirus prevalence reached 41.3%. Common genotypes included G1P[8], G9P[8], and G9P[4]. |
| ST37 | Eftekhari et al. (2023) | Iran | 200 | Norovirus | Norovirus prevalence was 20%. Genogroup GII dominated (95%), particularly GII.4 (53%). |
| ST38 | Othman et al. (2022) | Egypt | 50 | Multiple viruses | Viral detection rate was 90%. Norovirus GII (52%) most frequent followed by Rotavirus (24%). Coinfections occurred in 18%. |
| ST39 | Mathew et al. (2023) | Qatar | 231 | Rotavirus | G3 genotype dominant regardless of vaccination status; multiple G/P combinations identified. |
| ST40 | Alsubaiei et al. (2023) | Saudi Arabia | 92 | Multiple viruses | Rotavirus detected in 54%, Astrovirus 27%, Adenovirus 22.2%, Norovirus GI 9.5%, and Enterovirus 6.3%. |
| ST41 | Hijazi et al. (2022) | Qatar | 89 | Multiple viruses | Adenovirus most frequent pathogen, followed by Coxsackievirus A16, Rotavirus, Norovirus GII, Influenza A, and RSV A. |
| ST42 | Mashaly et al. (2022) | Egypt | 100 | Multiple viruses | Rotavirus detected in 43.9%, Norovirus in 36.8%, and coinfection in 15.8% of cases. |
| ST43 | Motamedi-Rad et al. (2020) | Iran | 361 | Rotavirus | Dominant genotype combination was G3P[8], with multiple G and P lineage diversity identified. |
